# The After-Clap

**Published:** 1894-12

**Authors:** 


### Editorial.

### THE AFTER-CLAP.

The surgeons of the latter part of the nineteenth century seem to have come to the conclusion that "the clap," as it is familiarly called, is not quite so harmless as a bad cold. The gynecologists tell us that a man should not marry who has ever had gonorrhœa, and they would certainly advise that no woman should marry a man who has had gonorrhœa. The evil effects of gonorrhœa seem to be far-reaching, indeed; for outside of the usual sequelæ, such as stricture, orchitis, epididymitis and gleet, we have gonorrhœal rheumatism, sterility, in both sexes; and pyosalpinx, and diseased tubes and ovaries in women.

The surgeon must begin to caution his patients about contracting the forerunner of these diseases, just as he used to do against placing the gonorrhœal virus in the eye, or against using an injection that is too strong.

There recently came under my observation a case of urethral arthritis, which presented all the characteristic symptoms of a specific origin, but which seemed to be unknown to the victim, whose wife had been away from him for some time. The temperature rose to 102°, and the pulse to 95, while the pain and swelling was confined, for the most part, to the wrist. He was, for nearly four weeks, unable to use his hand, and was under treatment for nearly five weeks. His case was a very stubborn one, but finally yielded to iodide of potassium and bichloride of mercury, taken internally, in large doses. His wrist was stiff for some time, but by the diligent use of a liniment of turpentine, camphor and oil, and of an ointment of compound iodine, belladonna, camphor and mercury, supplemented by bandages, cotton batting, and, finally, hot water, regained, to a great degree, its natural mobility.

A case of relative sterility, of over three years' standing, was recently presented to the students of the Southern Medical College, at the surgical clinic of Prof. Gaston. The history dated back to an attack of gonorrhœa. A negro man, aged 28, in good general health, had noticed something growing "on to his testicle," as he called it. The mass is now about the size of a cat's testicle, and is quite hard and unyielding, being continuous with the vas deferens and testicle. On the left side the prominence is more marked, but on the right side is a similar mass, but more elastic and yielding.

The spermatic cord is much more hardened on the right than on the left side, while the inguinal glands also show more enlargement on the right side.

The patient has the general appearance of a strong man, with clear eyes, and no enlargement of the post-cervical lymphatic glands. His lips showed slight abrasions, but he gives no specific history whatever. He is unusually intelligent for the ordinary black man. Upon being questioned, he gave the following account of his troubles:

From the time he first reached puberty, he began to have intercourse, never having any difficulty, either in finding willing partners to his act, or in ejaculating the semen, which seemed to him perfectly free. Three years ago he had a discharge of a yellowish color, which continued for some time, with very little treatment. He noticed that there was a scalding sensation upon urinating, but as there was no sore whatever, paid very little attention to it. Some doctor did, at one time, give him an injection, which he used for a short time. Later in the progress of the discharge, his testicle enlarged to the size of a pullet's or guinea egg, and one of the enlargements—that on the right side—opened and discharged a considerable amount of pus. The other gradually subsided, but a hard mass was left.

He has found that no semen passes upon intercourse, while he has no decrease, but rather an increase, of sexual desire. He is amply potent, and his penis is very large, with the foreskin well back of the sulcus of the glans.

The meatus urinarius is small for the circumference of his penis, but not smaller than No. 24. He was not examined for stricture, but, very likely, has a stricture of large calibre; as his gleety discharge continues.

A diagnosis of occlusion of the vasa deferentia from double epididymitis following gonorrhœa was made. It is a rare case, and Prof. F. W. McRae, who was asked to examine the case, states that he had never seen such a case, but concurred in the diagnosis. Prof. J. McF. Gaston was of the same opinion, and had never seen a case of total occulsion of the vasa deferentia before.

He stated to the class that the man might pass some semen, and yet not be able to ejaculate it. He also thought it possible that an operation of removing the diseased epididymis on one side might be of service. The patient was placed upon no internal treatment, but was ordered to apply locally the compound iodine ointment, and to report for an operation, if no improvement occurred.

